# Liquid biopsy in lymphoma: Is it primed for clinical translation?

**DOI:** 10.1002/jha2.212

**Published:** 2021-05-12

**Authors:** Edward Poynton, Jessica Okosun

**Affiliations:** ^1^ Centre for Haemato‐Oncology Barts Cancer Institute, Queen Mary University of London London UK

**Keywords:** biomarkers, circulating tumour DNA, liquid biopsy, lymphoma

## Abstract

The simultaneous growth in our understanding of lymphoma biology and the burgeoning therapeutic options has come with a renewed drive for precision‐based approaches and how best to incorporate them into contemporary and future patient care. In the hunt for accurate and sensitive biomarkers, liquid biopsies, particularly circulating tumour DNA, have come to the forefront as a promising tool in multiple cancer types including lymphomas, with considerable implications for clinical practice. Liquid biopsy analyses could supplement existing tissue biopsies with distinct advantages including the minimally invasive nature and the ease with which it can be repeated during a patient's clinical journey. Circulating tumour DNA (ctDNA) analyses has been and continues to be evaluated across lymphoma subtypes with potential applications as a diagnostic, disease monitoring and treatment selection tool. To make the leap into the clinic, these assays must demonstrate accuracy, reliability and a quick turnaround to be employed in the real‐time clinical management of lymphoma patients. Here, we review the available ctDNA assays and discuss key practical and technical issues around improving sensitivity. We then focus on their potential roles in several lymphoma subtypes exemplified by recent studies and provide a glimpse of different features that can be analysed beyond ctDNA.

## 1 INTRODUCTION

There have been significant strides in our understanding of lymphoma biology particularly with the advent of high‐resolution sequencing technologies. Through these approaches, cataloguing of the genetic landscape for the majority of lymphoma subtypes is nearing completion.[1–7] In parallel with this explosion in genetic information, the field has also witnessed a similar scale of expansion in the treatment armamentarium from conventional chemotherapy to novel targeted therapies like BTK inhibitors and next‐generation immunotherapies like checkpoint inhibitors and chimeric antigen receptor T‐cell therapy (CAR‐T).[8, 9, 10] With this, the focus in patient care is shifting to define how best clinicians can adopt refined precision‐based approaches in lymphoma management. Can we identify high‐risk patients at presentation or during initial treatment? Can we improve patient selection and predict who will respond and who will not to a defined therapy? Furthermore, the recent wealth of biological studies remind us that genomic alterations in lymphoma are dynamic, being acquired or lost temporally and/or spatially in response to a range of endogenous and exogenous selective pressures, including therapies, leading to complex tumour heterogeneity that eventually can contribute to therapy failure[11, 12, 13, 14, 15]. Technologies capable of capturing this heterogeneity throughout cancer development and progression are key to the success of precision approaches and have spurred the development of liquid biopsies, particularly circulating cell‐free DNA (cfDNA) as a tool that can serve as a real‐time surrogate measure of a patient's disease state.

The term liquid biopsy was first used to describe methods to derive diagnostic information about a tumoural lesion from a blood sample. The term is now used in a broader sense to refer to the sampling and analysis of analytes from various biological fluids, most commonly blood but also urine, ascites, cerebrospinal and pleural fluid, all of which are relatively straightforward to sample compared to a traditional tissue biopsy, the historical gold standard for cancer diagnosis. There are various analytes of interest within these liquid biopsy compartments including circulating tumour cells (CTCs); cfDNA; circulating cell‐free RNA (cfRNA), nucleosomes, extracellular vesicles (EVs); tumour‐educated platelets (TEPs); proteins; and metabolites[16]. As the majority of lymphoma patients present without circulating disease, much of the focus on the utility of liquid biopsies in lymphoma has been on the study of cfDNA which in patients with cancer is comprised of both circulating non‐tumour and tumour‐derived DNA (ctDNA) released by cells undergoing apoptosis and necrosis. CfDNA concentrations in healthy individuals range from 10 to 30 ng/ml but can be significantly higher in patients with cancer[17] and is influenced by a number of determinants including disease stage and metabolic tumour burden[18, 19]. In the case of lymphoma, there is additional variability by lymphoma subtype, with the highest levels of ctDNA seen in diffuse large B‐cell and primary mediastinal B‐cell lymphomas[20]. Additionally, plasma ctDNA concentrations are significantly lower in patients whose tumours are confined to the central nervous system (CNS) presumably due to the presence of an intact blood–brain barrier as an obstacle[21].

Given the minimally invasive nature of liquid biopsies and the ability for tumour‐derived ctDNA to serve as a better representation of the patient's lymphoma, the evaluation of ctDNA in lymphomas has significantly expanded in recent years. In this review, we begin by summarising the rationale of ctDNA analyses, currently one of the most intensively studied analytes in liquid biopsy samples. We review some of the factors that can influence the validity and sensitivity of ctDNA assays and detail its clinical utility in the context of lymphomas, and end with an outlook of what is to come in the not‐too‐distant future.

## 2 POTENTIAL BENEFIT: THE PLACE OF LIQUID BIOPSY

One of the main barriers to rapidly incorporating precision strategies in lymphoma is the issue of having sufficient tissue to guide clinical decision‐making. Although pathological confirmation with a biopsy has been the gold standard in lymphoma and remains indispensable for diagnosis, there remain some notwithstanding challenges. Surgical excision biopsies are frequently touted in lymphoma guidelines as the ideal for diagnostic histological confirmation but in practice, radiologically guided core or needle biopsies are becoming more commonplace due to the ease and speed by which these can be arranged. Once obtained, a typical requisite is for the tissue to be fixed with formalin and embedded in paraffin in preparation for histological analyses. These two factors limit both the quantity and quality of tissue available for biomarker analyses after routine diagnostic assessments. Many lymphomas demonstrate biological heterogeneity, both spatially and temporally[11, 12, 13, 14, 15]; however, multiple and/or repeat serial biopsies are rarely undertaken for the majority of patients. Therefore, molecular or biomarker analyses have become heavily reliant on a single diagnostic biopsy sample. Conventional imaging complements diagnostic tissue biopsies and is used for staging and response assessment but liquid biopsies may offer specific advantages over both tissue biopsies and imaging (**Table** **1**).

**TABLE 1:** Comparison of traditional tumour biopsy, radiological tumour assessment and ctDNA biomarker analysis

	**Tumour biopsy**	**Liquid biopsy–ctDNA**	**Imaging**
Accessibility	Invasive	Minimally invasive	Non‐invasive
Sampling risk	Non‐minimal, biopsy site dependent	Minimal	Minimal
Data interpretation	Requires experienced pathologist	Requires laboratory with genomics and bioinformatics capability	Requires experienced radiologist
Turnaround time	Days to weeks	Weeks to months	Hours to days
Relative cost	Sampling: Moderate costs Analysis: Low costs	Sampling: Low costs Analysis: High costs	Moderate costs
Potential clinical applications	Diagnosis Confirming relapse Molecular testing	Diagnosis (in specific settings) Response assessment MRD monitoring Clonal evolution	Staging Biopsy targeting Response assessment Follow up monitoring
Limitations	Tumour heterogeneity Inaccessible tumours Low‐quality nucleic acids	Lack of standardisation Sensitivity for MRD Patient‐specific panels costly	Radiation risk Lack of tumour specificity Not suitable for MRD

Abbreviation: MRD, minimal residual disease.

## 3 CONSIDERATIONS FOR ctDNA‐BASED ANALYSES

**FIGURE 1:**
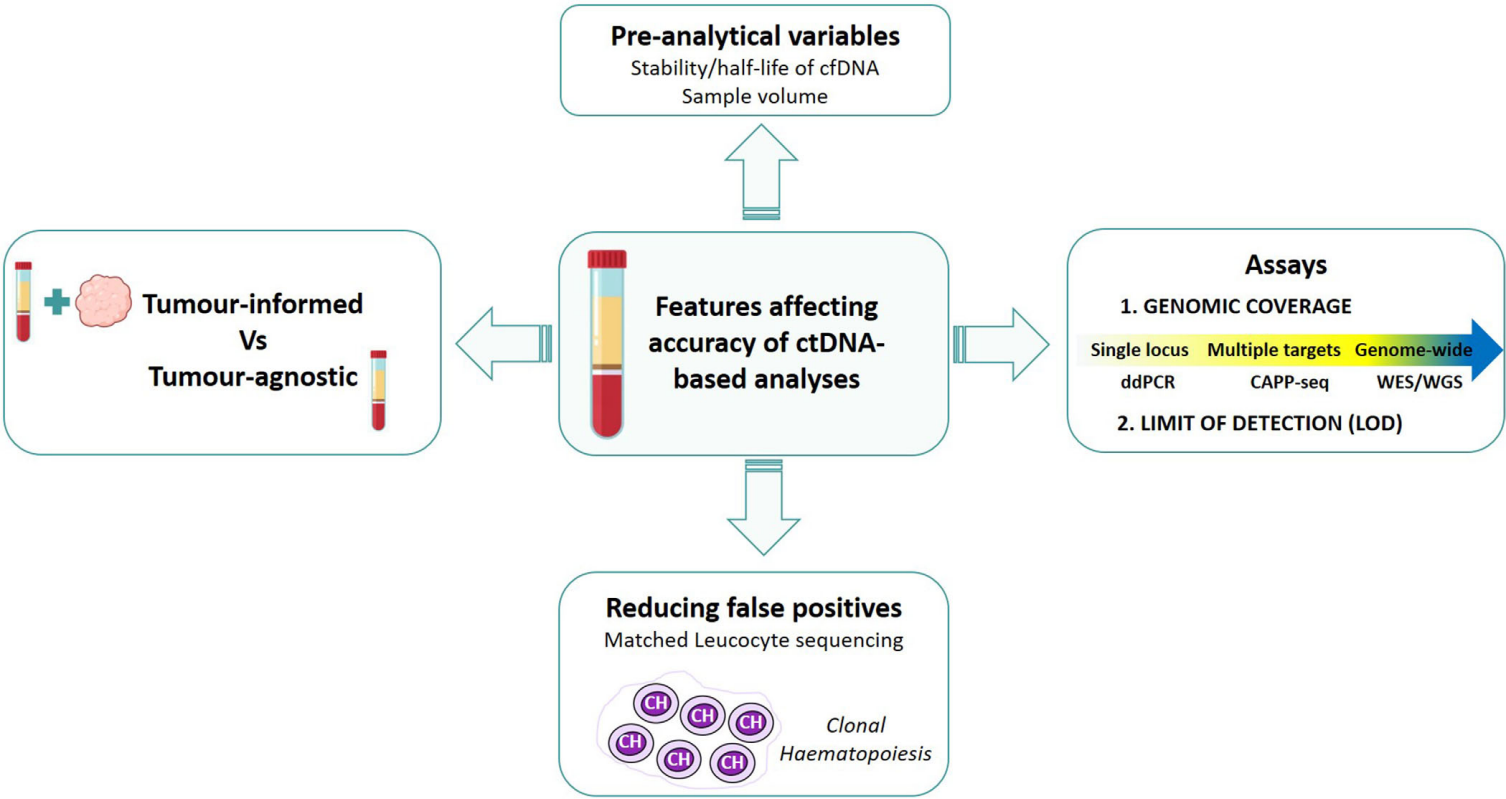
Features affecting the accuracy of ctDNA‐based analyses. A range of features ranging from preanalytical variables to type of assays to the inclusion or not of paired tumour and leucocyte (white blood cell) sequencing impact the sensitivity of the ctDNA analyses

Several factors can influence the sensitivity and, in turn, the clinical validity and utility of ctDNA‐based assays (**Figure** **1**). These include the pre‐analytical variables, technology and assay characteristics, whether a tumour‐informed or tumour‐agnostic approach is adopted, and the downstream bioinformatics analyses. As many of these are covered in many other comprehensive and excellent reviews[22, 23, 24], we will focus on those of most relevance to lymphoma ctDNA analyses.

### 3.1 Pre‐analytical variables

A number of variables need to be accounted for in order to best optimise sampling, collection and processing of ctDNA from blood draws or other liquid compartments. First, ctDNA typically constitutes a small proportion of an individual's cfDNA, which in turn is also present at low concentrations. For cfDNA isolation from peripheral blood, sampling of at least 10 ml of blood is recommended to ensure an adequate amount of cfDNA is isolated[25]. Second, ctDNA is very labile and is rapidly cleared from the circulation, with a half‐life of approximately 2 h. Last, there is the potential for contamination of samples by cfDNA released during normal immune cell lysis, which further dilutes the ctDNA concentration. While blood can be drawn into standard K_2_EDTA tubes, plasma isolation should be performed as soon as possible or at least within 4–6 h. The use of specialised cfDNA‐stabilising tubes (such as Streck cfDNA collection tubes) is preferable especially for multi‐centre studies as they extend the stability of cfDNA, allowing for processing to occur 2–7 days following collection. Additional care should be taken to optimise sample storage processes and avoid unnecessary freeze‐thaw cycles which hasten ctDNA degradation.

### 3.2 Assays and technologies

Given the potential low frequency of putative somatic gene variants against elevated levels of background noise, high sensitivity is a requisite for ctDNA assays. There are primarily two genotyping methods currently in use: polymerase chain reaction (PCR)‐based detection methods such as droplet digital PCR (ddPCR) and next‐generation sequencing (NGS)‐based methods. ddPCR offers high sensitivity (VAF ≤0.01%), is relatively straightforward to set up and has a quick turnaround without the need for complex bioinformatics analyses. However, as amplicons are limited to less than 1 kb (typically <200 bp), their utility is limited to the detection of known recurrent “hotspot mutations” (such as *MYD88* L265P mutations highly recurrent in lymphoplasmacytic lymphoma or *EZH2* Y641 mutations in diffuse large B‐cell and follicular lymphoma) and the assay has low multiplexing capabilities. In contrast, NGS allows for massively parallel sequencing of a large number of genetic loci simultaneously. As the maximum number of sequenceable base pairs is limited by the current NGS technology, increasing genomic coverage results in a decrease in the depth of sequencing achievable. One of the initial NGS‐based assay used in B‐cell lymphomas takes advantage of the unique biology of B‐ cell receptors. As each lymphoma comprises specific clonotypes defined by immunoglobulin VDJ rearrangements, these can be identified in lymphoma tissue through high throughput sequencing using universal primer sets (IgNGS, ClonoSeq) to encompass all possible rearrangements of the immunoglobulin loci. These pre‐determined tumour‐defined clonotypes are then trackable in the ctDNA of the patient. Due to the high sequencing depth required to accurately call low allele frequency variants, whole exome (WES) or genome sequencing (WGS) strategies run the risk of missing low‐frequency variants and therefore high depth sequencing of targeted panels of genes known to be recurrently mutated in lymphoma is more the norm. This is the basis of the cancer personalised profiling by deep sequencing (CAPP‐seq) approach that employs a comprehensive targeted gene panel design to detect lymphoma‐associated single nucleotide variants (SNVs), copy number alterations (CNAs) and structural variants (SVs)[26]. The design of the gene panel (also known as a selector) is tailored to the specific lymphoma and encompasses a range of gene loci known to be recurrently mutated within the lymphoma subtype. To improve the sequencing sensitivity, CAPP‐seq incorporates unique molecular identifiers (UMIs), molecular tags that facilitate downstream bioinformatic processing of sequences derived from the same DNA fragment (duplicates) and enable sequencing errors to be easily identified and excluded from subsequent analyses. As well as molecular barcoding, CAPP‐seq incorporates an in silico computational digital error suppression tool for further elimination of sequencing errors which improves the detection sensitivity to 1 mutant DNA molecule in 10,000 normal DNA[27]. There are now several different commercially available cfDNA library preparation assays although cross‐platform comparisons to standardise and optimise sensitivity and specificity thresholds are still lacking.

### 3.3 Roles of tumour and germline DNA

The ideal scenario is one where adequate tumour tissue is readily available in all patients so that the somatic variants are identified prior to or alongside ctDNA profiling to allow the identification of baseline tumour genotypes and a reference for bespoke longitudinal ctDNA surveillance, a so‐called *tumour‐informed approach*. In cases where paired tumour tissue is unavailable, a *tumour‐agnostic approach* where ctDNA analysis is performed without prior knowledge of a patient's tumour mutation profile can be employed. The advantages of the tumour‐agnostic approach are that profiling is less complex, associated with lower costs, faster turnaround time, and the ability to detect emerging resistant mutations. However, the trade‐off in the absence of the tumour genotype is a higher rate of false positives and the inability to track a known tumour VDJ clonotype. Though more resource‐intense, a tumour‐informed approach aids in identifying false negatives in ctDNA and is thus particularly well‐suited for broader minimal residual disease (MRD) detection (where accurate calling of low‐frequency variants is essential) and disease recurrence monitoring.

Another consideration is the inclusion of germline DNA such as derived from saliva or buccal swabs to identify variants that are truly somatic in nature. As normal haematopoietic cells accumulate somatic mutations during ageing which can drive clonal expansions of haematopoietic cells, referred to as clonal haematopoiesis (CH)[28, 29] which acts as a potential confounder, particularly in tumour‐agnostic approaches. Studies have demonstrated that a high prevalence of somatic variants in cfDNA (>50%) in both cancer patients and healthy individuals can be attributed to clonal haematopoiesis[30, 31, 32]. These CH‐related variants can be filtered out using novel bioinformatics approaches or by sequencing matched cfDNA and leucocyte‐derived DNA in parallel to aid identification and elimination of false‐positive, CH‐specific variants[30, 31].

## 4 APPLICATIONS IN SPECIFIC LYMPHOMA SUBTYPES

### 4.1 Diffuse large B‐cell lymphoma

Diffuse large B‐cell lymphoma (DLBCL) is the most common high‐grade lymphoma and exhibits marked biological and clinical heterogeneity[33]. Over 20 years ago, DLBCL was broadly classified into two binary subtypes (germinal centre and activated B‐cell) based on the cell of origin (COO) determined by gene expression[34]. The recurrent genetic landscape of DLBCL has been extensively studied[1–3, 35]. More recently, multiple distinct subtypes of DLBCL based on gene mutations and copy number alterations have been identified through genome‐wide profiling suggesting the dawn of a new molecular taxonomy system[36, 37, 38]. Five‐year overall survival in DLBCL is approaching 70%^33^ and although approximately 50% of patients can be cured with induction treatment alone, patients who relapse or are refractory to salvage or autologous stem cell transplantation have a very poor prognosis with 2‐year overall survival from relapse of around 20%[39]. Whilst the emergence of therapies like CAR‐T cell therapy[40, 41, 42] and bispecific T cell antibodies are promising, at present, there are few curative treatment options for the majority of patients in this poor‐risk group. Prognostic tools such as the international prognostic index (IPI) and COO have little impact on treatment decisions. Imaging modalities, such as fluorodeoxyglucose (FDG) positron emission tomography (PET)/computed tomography (CT) are used both as part of the diagnostic workup and as response assessment tools following treatment. Although, interim PET‐CT may have prognostic relevance, parameters such as total metabolic tumour volume (TMTV) and standardised uptake value (SUV) have varying predictive utility[43, 44] and interim analyses with intensification has not shown demonstrably improved outcomes[45, 46].

Among all lymphoma subtypes, applications of liquid biopsy have been best examined in DLBCL. The rarity of circulating tumour cells in aggressive lymphomas has sparked the investigation of additional liquid biopsy analytes, principally ctDNA in DLBCL. Both plasma‐derived and serum‐derived cfDNA has been studied in DLBCL and although higher concentrations of total DNA can be isolated from serum samples, cfDNA is contaminated by high fragment length genomic DNA released by lysing cells during in vitro sample preparation and thus plasma has become the specimen of choice for peripheral blood ctDNA analysis[47]. Initial studies leveraged the ability to identify tumour‐specific Ig clonotypes in approximately 80% of pre‐treatment samples and demonstrated the feasibility of tracking these clonotypes in serum and plasma ctDNA during a patient's disease course[48, 49]. Subsequent studies began adopting the targeted gene panel CAPP‐seq strategy so that multiple somatic variants can be tracked longitudinally in each DLBCL patient, thus overcoming the limitations of tracking single Ig clonotypes[48]. Unsurprisingly, ctDNA levels at diagnosis corresponded with radiological and blood‐based surrogates (LDH) of tumour burden and higher pre‐treatment ctDNA levels were independently associated with inferior progression‐free survival[48, 50, 51]. Perhaps the most value in DLBCL treatment is getting an early gauge on those who will best versus least respond to standard induction treatments. Detectable ctDNA levels, using Ig clonotypes, following two cycles of induction treatment was associated with a much shorter time to progression compared to those without (42% (CI 22.2 to 60.1%) versus 80% (CI: 69.6 to 87.3%) at 5 years (*p* < 0.0001)[48]. Additionally, using sequential surveillance ctDNA monitoring, in patients who have overt relapses following induction therapy, ctDNA levels were detectable several months prior to radiological evidence of disease relapse[48, 50]. With the CAPP‐seq strategy for dynamic ctDNA monitoring, Kurtz and colleagues demonstrated that not only was achieving undetectable ctDNA levels at the end of induction treatment prognostic but attaining at least a 2‐log fold reduction after one cycle and 2.5‐log fold reduction after two cycles of induction treatment conferred a superior event‐free survival advantage at 24 months[52].

These studies convincingly demonstrate a plausible role for ctDNA in follow up and post‐treatment MRD monitoring and risk stratification in DLBCL. A rather fascinating study by Kurtz and colleagues highlights the potential of an approach utilising dynamic risk assessment whereby a continuous individualised risk index (CIRI) based on a Bayesian statistical model is calculated by considering both pre‐treatment and dynamic on‐treatment risk factors in order to estimate outcomes at any given time point during a patient's disease or treatment journey[53]. How these iterative real‐time analyses will shape how we modulate treatment during a patient's disease course will only be known over time with additional large‐scale prospective studies.

The earliest studies investigating ctDNA in DLBCL established that plasma ctDNA could serve as a good surrogate to tumour biopsies with a high concordance between the genotype of diagnostic biopsies and plasma ctDNA. This is relevant given the challenges and infrequency of repeat biopsies, especially in the context of relapsed or refractory DLBCL (rrDLBCL). By performing targeted gene panel sequencing of primarily the plasma ctDNA in over 100 patients with rrDLBCL, Rushton and colleagues demonstrated differences in the mutational profiles between diagnostic and rrDLBCLs, with enrichment in *KMT2D* and *TP53* mutations at progression[54]. It also appears feasible to utilise plasma ctDNA for classifying DLBCL into its different molecular entities[55].

Given the broadening gamut of therapies, particularly in the rrDLBCL setting, predictive biomarkers and response assessment using ctDNA is an important biomarker opportunity. Droplet digital PCR for tracking a number of recurrent actionable hotspot mutations such as *EZH2* Y641N and *MYD88* L265P may have utility in identifying those who will benefit from specific therapies and in monitoring responses[56, 57], although it is unlikely to be suitable for capturing emerging broader resistance mechanisms. CD19 CAR‐T therapy has transformed the treatment landscape for rrDLBCL[40, 41, 42] with durable remissions in approximately 45–50% of patients[58]. Crucially, it is clear that these therapies do not work in every patient, are expensive and associated with important short‐ and long‐term toxicities. Ascertaining predictive determinants that drive response and failures will aid in improve patient selection. In this setting, minimally‐invasive Ig clonotypes or non‐Ig mutations tracked in ctDNA as an MRD assessment strategy following CD19 CAR‐T therapy showed that interim ctDNA levels as early as 7 and 28 days had prognostic relevance[59, 60].

### 4.2 CNS lymphoma

Cancers that affect the CNS pose a particular challenge because of the invasiveness and difficulty in safely accessing tissue for diagnosis. Due to anatomical proximity, ctDNA can be more readily detected in CSF‐derived cfDNA from CNS lymphomas, primary and secondary brain cancers[61, 62] than plasma‐derived cfDNA[63]. Primary CNS lymphoma (PCNSL) is a rare and clinically aggressive lymphoma subtype accounting for about 4% of all brain tumours. Although methotrexate‐based chemotherapy that crosses the blood–brain barrier is effective at induction[64], it is challenging to deliver in older patients and altogether there remains a significant proportion of patients who experience early relapses[65]. The utility of ctDNA in PCNSL is less well studied than in systemic DLBCL but studies have demonstrated that CSF‐derived ctDNA detection is more sensitive than conventional flow cytometry for the detection of PCNSL recurrence and secondary CNS involvement in systemic DLBCL[66].

The earliest studies evaluating the utility of ctDNA focussed on the *MYD88* L265P mutation due to its presence in greater than 80% of PCNSL tumours. *MYD88* mutations are exceptionally rare in non‐lymphoma CNS malignancies and thus could be used to distinguish from PCNSL. The sensitivity in detecting *MYD88* mutations in plasma‐derived ctDNA has varied in different series. In one study, *MYD88* mutations were identified in approximately one‐third of PCNSL cases that harboured the mutation within the tumour sample, with considerably lower variant allelic fractions (VAFs) in the ctDNA compared to tumour DNA[67]; although ctDNA was detectable in about 50–60% of patients in another small series of 14 patients[68]. Watanabe and colleagues used a ddPCR approach to demonstrate *MYD88* L265P mutations were detectable in CSF from 20/26 cases of CNS lymphoma[69] suggesting that the CSF provides a more reliable source of ctDNA to track PCNSL tumour‐specific mutations than plasma but one must bear in mind that lumbar punctures are certainly more invasive than a routine blood draw.

Secondary CNS lymphoma (SCNSL) is a rare but devastating complication of systemic DLBCL and as such, there have been numerous efforts to identify patients with a low and high risk of CNS relapse in order to rationalise the use of toxic CNS‐penetrating chemotherapy regimen. The majority of CNS relapse prognostication tools rely on clinical parameters such as the CNS‐IPI[70]. In a recently published study, ddPCR is used to identify tumour‐defining mutations in CSF derived ctDNA. Here, the authors detected mutations in all PCNSL cases (*n* = 6), one case of systemic DLBCL with subsequent SCNSL involvement but in no cases of DLBCL without CNS involvement (*n* = 12)[66] indicating ctDNA levels in CSF could be used to predict the risk of CNS relapse. It also appears that higher cfDNA concentrations and mutations in CSF‐derived ctDNA in just five genes (*BTG2*, *PIM1*, *DUSP2*, *ETV6* and *CXCR4*) were associated with high‐risk CNS IPI scores[71]. These pilot studies tantalisingly hint at the possibility of using ctDNA to strengthen existing risk‐prediction tools, although evaluation in larger scale studies is warranted.

### 4.3 Intravascular large B‐cell lymphoma (IVLBCL)

Intravascular large B‐cell lymphoma (IVLBCL) is a rare subtype of extranodal aggressive B‐cell lymphoma that is peculiarly confined, although not exclusively, to the lumen of blood vessels (intravascular space) without lymphadenopathy, making diagnosis extremely challenging. Due to the diffuse nature of the lymphoma infiltrate and lack of tumour masses, IVLBCLs are difficult to detect with conventional imaging and are often missed by tissue biopsy leading to delays in diagnosis and poor patient outcomes, highlighting an opportunity for ctDNA analyses to aid diagnoses. Recent data suggest that ctDNA levels may be higher in IVLBCL compared to DLBCL[72]. In a series of nine cases of IVLBCL, targeted sequencing of diagnostic time‐point plasma or serum cfDNA with an 8‐gene panel could demonstrate at least one recurrent somatic variant in every patient with notably more mutations and significantly higher VAFs identified in the cfDNA compared to the tumour tissue[73]. In the same study, the authors showed that specific mutations could be followed during the patient's disease journey and correlated with response and imminent relapse. Leveraging the high ctDNA levels in IVLBCLs, Shimada and colleagues undertook ctDNA WES in 18 patients to better define the mutational landscape of this rare entity, showing the presence of genetic lesions typically associated with ABC‐DLBCL (including *CD79B* and *MYD88)* but also showing recurrent rearrangements in the checkpoint ligands PD‐L1/PD‐L2^72^.

### 4.4 Hodgkin lymphoma

A detailed understanding of the genomic landscape in classical Hodgkin lymphoma (cHL) had lagged behind other B cell lymphomas owing to the rarity of Reed‐Sternberg cells (1–2%) within cHL biopsies[74, 75, 76]. The treatment of cHL has embraced a response adapted therapy approach, primarily driven by imaging‐guided (PET) interval response assessment to prognosticate which patients require treatment escalation and those in whom further treatment can be safely omitted[77]. Recent studies have begun to evaluate the role of ctDNA in cHL. In a cohort of 60 cHL patient diagnostic plasma samples, Camus and colleagues used a 9‐gene amplicon‐based NGS targeted panel to identify somatic variants. They found a higher variant allele frequency in ctDNA than in tumour biopsy derived DNA in 70% of cases, likely explained by the scarcity of RS cells within cHL biopsies[78]. Higher ctDNA concentrations correlated with advanced‐stage disease and higher baseline tumour metabolic volume. Among patients with paired diagnostic and follow up ctDNA samples in this study, no patients had detectable ctDNA variants after two cycles of standard chemotherapy (*n* = 31). However, some patients did go on to relapse at a later stage indicating that, at least in this study, ctDNA genotyping is highly specific but lacks sufficient sensitivity for use as an MRD tool although this could in part have been due to the limited gene panel and assay sensitivity.

In a prior study with a 77‐gene CAPP‐seq based targeted panel, the authors demonstrated a high concordance between variants present in ctDNA and tumour DNA (nearly 90%). In a cohort of 24 advanced‐stage cHL patients, a 2‐log fold reduction in ctDNA levels following two cycles of ABVD chemotherapy was associated with a PFS and overall survival advantage[79], findings akin to those reported in DLBCL[50].

### 4.5 Follicular lymphoma

Follicular lymphoma (FL) is the most common indolent NHL with heterogeneity in clinical behaviour. Early‐stage FL can achieve long remissions in about half of the patients with standard treatment modalities. For advanced‐stage FL, a subset can initially be managed by active monitoring, others may require multiple lines of therapy and there remains a high‐risk group who relapse early or transform to DLBCL and have significantly inferior outcomes[80]. Accurately risk‐stratifying patients and, particularly, identifying those with high‐risk behaviour remains an unmet need in FL. On the one hand, the concept and evaluation of MRD have had the longest history in FL due to the availability of a molecular marker, the BCL2‐IGH rearrangement. However, the detection and monitoring of the rearrangement are impacted by high rates of somatic hypermutation, variability of the breakpoint region and its presence in healthy individuals meaning that it can only be monitored in approximately 50–60% of patients[81]. Despite this, there continues to be interest and debate on the value of MRD monitoring for prognostic purposes in FL. The data on the role of ctDNA analyses in follicular lymphoma is slowly emerging. Notably, the quantifiable ctDNA levels are comparably lower in FL compared to aggressive lymphomas perhaps pointing to different kinetics and dynamics of the shedding from tumour cells[20].

In an initial series of 34 patients from the PRIMA trial, using clonoSEQ, VDJ clonotypes were identified in the plasma of 74% of patients, with a high ctDNA level at diagnoses corresponding with a significantly shorter PFS[82] providing a preliminary assessment on the feasibility of analysing these components within the plasma of FL patients. Like BCL2‐IGH rearrangements, VDJ clonotypes cannot be identified and tracked in every FL patient. In another study, baseline levels of cfDNA in 61 FL patients, measured by ddPCR approach, correlated with tumour burden measured by TMTV and higher cfDNA levels were associated with shorter 4‐year PFS[83].

The mutational landscape of FL, like many other lymphomas, is well defined[7, 13, 84, 85] and could be amenable to mutation‐based ctDNA tracking. In four patients with *EZH2* mutations detected by ddPCR in ctDNA, the *EZH2* VAFs fluctuated in accordance with tumour burden and in response to immunochemotherapy[86]. This may be valuable as a predictive biomarker to monitor responses to treatments such as tazemetostat, a small molecule EZH2 inhibitor, that has recently been approved in relapsed/refractory FL[87].

The capability to predict FL transformation is an area of interest due to adverse outcomes. In an early pilot series, transformation‐specific mutations within the tumour could be identified in the plasma ctDNA several months before the onset of clinical transformation[50], pointing to the potential of utilising this modality in tracking clonal evolution and capturing the known spatial and longitudinal heterogeneity in FL[7, 13, 14, 50, 84, 85].

### 4.6 Other lymphomas

There have been small but insightful studies in other B‐ and T‐cell lymphoma subtypes. In mantle cell lymphoma (MCL), Agarwal and colleagues showed that ctDNA genotyping results were non‐inferior to traditional flow cytometry or allele‐specific PCR MRD detection methods. Mutations in genes related to chromatin remodelling were associated with treatment resistance to ibrutinib and venetoclax and could easily be tracked in plasma ctDNA samples[88].

There is likely to be a tremendous benefit in T‐cell lymphomas as these subtypes more commonly are associated with extra‐nodal disease, which can at times be challenging to re‐biopsy. Plasma EBV‐DNA measurement has been used in post‐transplant lymphoproliferative disorder (PTLD) and extranodal NK‐T cell lymphoma (ENKTCL) as a diagnostic and disease‐monitoring tool[89]. In ENKTCL, higher levels of plasma EBV‐DNA at diagnosis are associated with a poorer outcome[90].

In a series of 65 patients with ENKTCL, there was over 90% concordance between plasma‐derived ctDNA with tumour tissue genotyping and certain mutated genes including *KMT2D* were associated with advanced stage and higher tumour metabolic volume[91]. Another study in 9 patients with PTLD showed detectable ctDNA levels prior to diagnosis and in the EBV‐positive patients, there was concordance with EBV‐DNA titres[92]. These studies raise the question of whether a combined approach using ctDNA analyses in tandem with EBV‐DNA titres may allow early detection in cases of PTLD and more accurately assess response following treatment.

Compared to B‐cell lymphomas, studies evaluating the feasibility of ctDNA analyses in other T cell lymphomas have thus far been scarce and in small cohorts, primarily of angioimmunoblastic and other peripheral T‐cell lymphomas[93, 94]. Many applications of ctDNA in B‐cell lymphomas will have a place in T cell lymphomas and in time, one anticipates an increasing number of studies with continued research in this space.

## 5 WHERE ARE THE PRIORITY AREAS FOR ctDNA IN LYMPHOMA CLINICAL PRACTICE?

**FIGURE 2:**
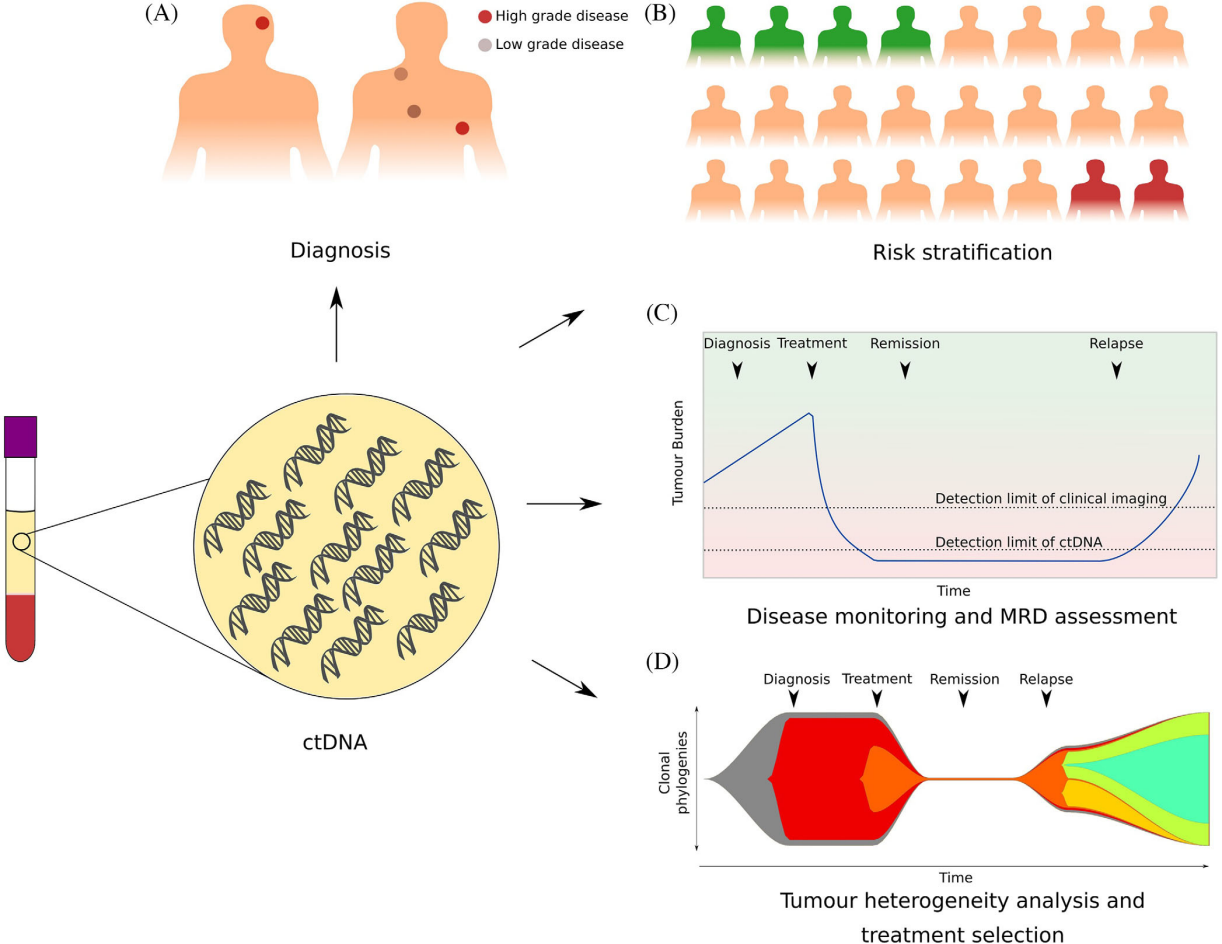
Potential applications for ctDNA analysis in the clinical management of lymphoma. (A) As a diagnostic tool in difficult to access tumour, identifying relapses or high‐grade transformation. (B) Risk stratification of patients into good‐ and poor‐risk groups to guide treatment selection. (C) Minimal residual disease (MRD) monitoring of patients following treatment allowing relapse detection at an earlier time point than is possible with conventional methods. (D) Detecting the emergence of treatment‐resistant subclones in order to guide treatment escalation decisions

The potential clinical applications of ctDNA range from aiding diagnoses to treatment selection and response and progression monitoring (**Figure** **2**). Altogether, the studies to date across the lymphoma subtypes have focussed on demonstrating concordance between ctDNA‐ and tissue‐based genotypes, examining how ctDNA correlates with clinical features and standard imaging surveillance and its utility as an interim assessment tool. There remain open areas that need further exploration:

1. Broad applicability: Larger studies will be required to assess the feasibility and suitability of these assays across the lymphoma subtypes.
2. Dual prediction and prognostication: In addition to comparisons with standard response assessment tools like imaging, the next steps would entail investigating whether these tools have sufficient predictive and prognostic accuracy to inform de‐escalation or escalation of therapy (for example maintenance or consolidation) as well as the selection of novel therapeutic strategies.
3. Molecular‐based endpoints: Can we envisage a future of defining remission molecularly in lymphoma? This would require the development and evaluation of more sensitive liquid biopsy assays to measure and monitor MRD.
4. Approved ctDNA‐based assays: For the clinical adoption of ctDNA assays, a detailed roadmap from careful evaluation and standardisation of the processes and methodologies to establishing utility, reproducibility and cost‐effectiveness is required[25].

## 6 MOVING BEYOND ctDNA GENOTYPING

Aside from quantifying and genotyping ctDNA, there is an emerging multitude of alternative ctDNA and non‐ctDNA parameters through which to derive additional information about the underlying tumour.

Current ctDNA profiling approaches focus on the high depth sequencing of a single locus or well‐defined targeted gene panel analyses. However, there are limitations to this strategy especially in the setting of evaluating low burden disease settings such as MRD that is of relevance in lymphomas. As the tumour burden is significantly lower at MRD states, there is a ceiling beyond which a higher depth of sequencing and complex bioinformatics analyses will not achieve the necessary resolution and sensitivity due to the low number of tumour‐specific DNA molecules in this context. Alternative approaches are being explored, whereby broader genome‐wide profiling using whole‐genome sequencing coupled with machine learning to track multiple genetic aberrations from mutations to copy number alterations at much lower sequencing depths could mitigate some of the challenges of restricted gene panel sequencing[95].

Beyond genetic changes, epigenetic deregulation is now a recognised hallmark of both B‐ and T‐cell lymphomas. DNA methylation of CpG sites is an important epigenetic regulator of gene expression with different tumour types exhibiting specific methylation signatures. Tumour‐specific methylation signatures can be detected in cfDNA and have been used to trace the tissue of origin of cfDNA or indeed to directly detect cancers with remarkable sensitivity and specificity[96, 97]. Using a technique referred to as cell‐free methylated DNA immunoprecipitation and high‐throughput sequencing (cfMeDIP‐Seq), Nassiri and colleagues demonstrated that extracted methylation signatures from just the top 300 differentially methylated regions (DMRs) could be used together with machine learning algorithms to distinguish glioblastoma from other brain tumours with a high degree of sensitivity and specificity[98]. This approach has also been applied to ctDNA from urine to detect early‐stage renal cell carcinoma[99]. In lymphoma, one study demonstrated that global hypomethylation by using a single methylation target, LINE1 as a surrogate, was associated with poor prognosis in DLBCL. Interestingly, LINE1 methylation could also be detected in the patient's cfDNA and was an independent prognostic factor[100]. Although this analysis was limited to a small number of dysregulated sites as opposed to the high throughput profiling which would be needed to identify the tumour‐specific methylation patterns, it perhaps highlights a potential avenue for further exploration in lymphomas.

Another evaluable and informative feature of ctDNA is the pattern of cfDNA length and fragmentation. It is known that cfDNA fragments roughly correspond to the length of DNA wrapped around a nucleosome (∼147 bp) plus linker DNA. Tumour derived ctDNA fragments have been shown to have greater variability and typically are distinctly shorter than non‐tumour derived cfDNA, in the region of 90–150 bp[101, 102]. These fragment size distribution differences are being leveraged to classify solid organ malignancies with a high degree of accuracy[100] and would have huge potential for early detection and monitoring of cancers. Additionally, the ability to selectively enrich for specific fragment sizes within the tumour ctDNA range would be advantageous to improve the resolution of ctDNA genotyping.

As cfDNA is predominantly released by apoptotic cells, it circulates in the form of nucleosome‐protected DNA fragments in the circulation. As nucleosome positioning is an epigenetic determinant of gene expression that is cell or tissue specific, studies have demonstrated that gene expression could be inferred from the differences in the nucleosome patterns between highly versus lowly/silently expressed genes (detected as differences in the sequencing depth at transcription start sites upstream of the genes) and this in turn could be used to determine the cell or tissue‐of‐origin[103, 104]. Notably, there has been one study in DLBCL where nucleosome mapping via targeted cfDNA profiling was used to determine the cell‐of‐origin[105].

There are a growing number of features and additional analytes including EVs, ctRNA, proteins and other components that are being explored in various cancer types that are beyond the scope of this current review. Many of these have yet to be thoroughly evaluated in lymphomas, although in time they may demonstrate tremendous added potential in combination with ctDNA genotyping.

## 7 CONCLUSION

Our understanding of ctDNA and its dynamics during the lymphoma disease course from diagnoses through treatment to progression has rapidly accelerated in a relatively short period of time and continues to expand. Published studies on the potential utility of ctDNA assessment in lymphomas have been in relatively small patient cohorts and there is a need for more detailed evaluations in much larger contemporary patient cohorts across the breadth of lymphoma subtypes. There remains a need for rigorous standardisation, harmonisation, and quality assurance processes together with the development of adequately sensitive assays especially in the setting of MRD. The current wave of clinical trials with integrated ctDNA profiling in lymphoma will be invaluable in demonstrating the feasibility, cost effectiveness, and utility in eventually bringing liquid biopsy analyses into our routine clinical workflows.

## AUTHOR CONTRIBUTIONS

Edward Poynton and Jessica Okosun reviewed the literature, wrote the manuscript and approved the final version.

## CONFLICT OF INTEREST

The authors declare that they have no conflict of interest.
